# Readability of Patient Education Materials on the American Association for Surgery of Trauma Website

**DOI:** 10.5812/atr.18161

**Published:** 2014-04-30

**Authors:** Adam E. M. Eltorai, Soha Ghanian, Charles A. Adams, Christopher T. Born, Alan H. Daniels

**Affiliations:** 1 Warren Alpert Medical School, Brown University, Providence, USA; 2 Division of Trauma and Surgical Critical Care, Department of Surgery, Rhode Island Hospital, Providence, USA; 3 Division of Orthopaedic Trauma, Department of Orthopaedic Surgery, Rhode Island Hospital, Providence, USA

**Keywords:** Patient Education Materials, Online Health Information, Readability, Comprehension, Flesch-Kincaid Formula

## Abstract

**Background:**

Because the quality of information on the Internet is of dubious worth, many patients seek out reliable expert sources. As per the American Medical Association (AMA) and the National Institutes of Health (NIH) recommendations, readability of patient education materials should not exceed a sixth-grade reading level. The average reading skill of U.S. adults is at the eighth-grade level.

**Objectives:**

This study evaluates whether a recognized source of expert content, the American Association for Surgery of Trauma (AAST) website’s patient education materials, recommended readability guidelines for medical information.

**Materials and Methods:**

Using the well-validated Flesch-Kincaid formula to analyze grade level readability, we evaluated the readability of all 16 of the publicly-accessible entries within the patient education section of the AAST website.

**Results:**

Mean ± SD grade level readability was 10.9 ± 1.8 for all the articles. All but one of the articles had a readability score above the sixth-grade level. Readability of the articles exceeded the maximum recommended level by an average of 4.9 grade levels (95% confidence interval, 4.0-5.8; P < 0.0001). Readability of the articles exceeded the eighth-grade level by an average of 2.9 grade levels (95% confidence interval, 2.0-3.8; P < 0.0001). Only one of the articles had a readability score below the eighth-grade level.

**Conclusions:**

The AAST’s online patient education materials may be of limited utility to many patients, as the readability of the information exceeds the average reading skill level of adults in the U.S. Lack of patient comprehension represents a discrepancy that is not in accordance with the goals of the AAST’s objectives for its patient education efforts.

## 1. Background

Health literacy is the ‘‘capacity to obtain, interpret, and understand basic health information and services and the competence to use such information and services to enhance health’’ (1). An individual’s health literacy is an independent predictor of their health-related quality of life (2) with low health literacy being associated with worse overall health (3), increased hospitalizations (4), increased complications that require hospital attention (3), poor understanding of one’s disease (5), and an overall increase in health-care costs (6).

Patients are increasingly using the internet to acquire health information (7, 8) with over eight million Americans seeking health information from the internet every day (7). However, the ability to utilize online health information to make healthcare decisions depends on the ability to comprehend the material (8). As an expert website, the American Association for Surgery of Trauma (AAST) has an obligation to make sure its content is not only accurate, but also readable.

The reading comprehension level determines the readability that a text must have so that a reader understand the written materials (9). The Flesch-Kincaid Grade Level (FKGL) formula is a validated and common instrument used to determine the readability of written materials in terms of the United States academic grade levels (10-18). The FKGL formula was originally used by the United States Army for assessing the difficulty of technical manuals and became the Department of Defense Military Standard. The higher the FKGL of a text, the more difficult it is to read and comprehend, requiring more advanced reading skills. 

The average American adult reads at an eighth-grade level (19, 20). Approximately, 47% of adults in the United States “experience considerable difficulty in performing tasks that required them to integrate or synthesize information from complex or lengthy texts” (21). Nearly one-fifth of adults in America cannot comprehend fourth-grade-level text (20). Doak and Doak investigated the reading level of patients at a public hospital, and found the average patient read at a seventh-grade level, despite having reported high school graduation (22).

Organizations including the American Medical Association (AMA) and the National Institutes of Health (NIH) recommend that the readability of patient education materials should be no greater than a sixth-grade reading level (23-27). Despite these recommendations, several studies suggested that current patient education materials might be at a reading level that is too complex for most patients to comprehend (10, 11, 13, 14, 16, 18, 20, 28).

## 2. Objectives

To our knowledge, the readability of the online patient information on the AAST website had not previously been assessed. Therefore, the goal of this study was to evaluate the readability of patient education materials on the publicly available AAST website to determine whether it met recommended readability guidelines for medical information. We hypothesized that the readability of these online materials would be a FKGL of > 6.

## 3. Materials and Methods

This study analyzed the patient education materials on the AAST website (http://www.aast.org/GeneralInterest/Links.aspx). The study was exempt from Institutional Review Board (IRB) review. The website is publicly accessible and was accessed on October 23, 2013. All 16 patient education articles were assessed for this study. No participants were recruited for this study.

Text from the website was copied in plain text format into individual Microsoft Office Word 2010 (Microsoft Corporation, Redmond, WA, USA) documents. Copyright notes, date stamps, author information, hyperlinks, citations, tables, and any other text not directly related to patient education were deleted. To avoid underestimating the readability level, numbers, decimals, bullets, abbreviations, paragraph breaks, colons, semicolons, and dashes within a sentence were removed, as recommended by Flesch and others (29, 30).

The FKGLs were obtained for each document by using the readability calculator built into the Word. The FKGL is calculated by the following formula: 

[0.39 × (average number of words per sentence)] + [11.8 × (average number of syllables per word)] − 15.59.

Sequentially selecting “File, Options, Proofing, and Show readability statistics” enabled the built-in FKGL calculator on Microsoft Word. The FKGL for each document was automatically displayed after grammar and spelling were checked. Each FKGL was recorded. It was more convenient to use the Word version of the FKGL calculator since the text that was being analyzed was already copied and pasted into a Word document. To compare the mean FKGL with the recommended readability level (the AMA and NIH as well as the average American adult reading level), an unpaired t-test was used. A statistical cutoff of P <0.05 was used for the determination of significance.

## 4. Results

**Figure 1.**
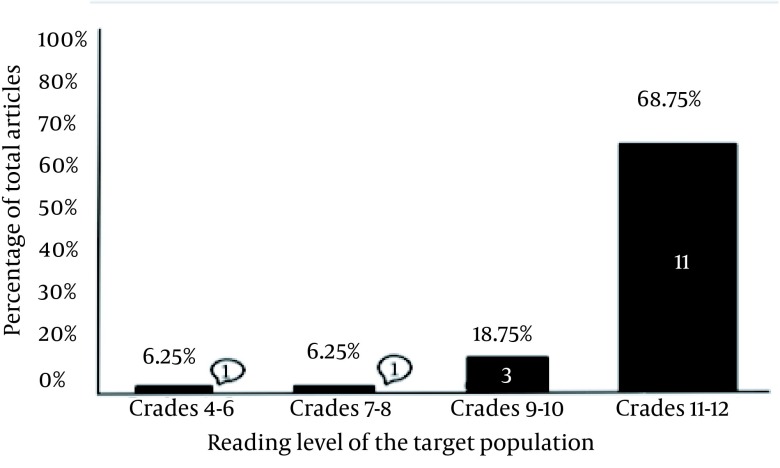
The Flesch-Kincaid Grade Levels of Patient Education Articles Available on the American Association for Surgery of Trauma (AAST) Website Eleven of the 16 articles had a readability grade level between 11 and 12, three of the articles had a readability level between nine and ten, one of the articles had a readability level between seven and eight, and only one of the articles had a readability level between four and six.

The mean readability of all 16 patient information pages was grade level 10.9±1.8. Except one, the rest of the articles (93.8%) had a readability score above the sixth-grade level, the maximum level recommended by the AMA and the NIH. The readability of the articles exceeded this level by an average of 4.9 grade levels (95% CI: 4.0-5.8; P < 0.0001) (Table 1). Only one of the articles had a readability score below the eight-grade level, the average reading skill level of the United States adults. The readability of the articles exceeded this level by an average of 2.9 grade levels (95% confidence interval, 2.0-3.8; P < 0.0001) (Figure 1).

**Table 1.** The Flesch-Kincaid Grade Level and Levels Above Sixth-Grade for Each Analyzed Article ^a^

Article Title	Readability Grade Level (FKGL)	Grade Levels Above Recommended (FKGL–6.0)
**“Aspiration in Trauma”**	12.0	6.0
**“Blunt Cardiac Injury”**	12.0	6.0
**“Blunt Splenic Trauma”**	12.0	6.0
**“Child Passenger Safety”**	8.5	2.5
**“Cost of Injury”**	12.0	6.0
**“Critical Care Illness”**	12.0	6.0
**“Discharge Instructions for Wound Care”**	6.0	0.0
**“Epidemiology and Injury Prevention”**	11.9	5.9
**“Field Triage of the Injured Patient”**	12.0	6.0
**“Mechanical Ventilation in the Intensive Care Unit”**	12.0	6.0
**“Pelvis Injuries”**	10.4	4.4
**“Rib Fractures”**	10.9	4.9
**“Sports Concussions”**	9.4	3.4
**“Thromboembolic Disease”**	12.0	6.0
**“Trauma Systems”**	11.0	5.0
**“Traumatic Brain Injury Rehabilitation”**	12.0	6.0
**Mean**	10.9	5.0

^a^ Abbreviations: FKGL, Flesch-Kincaid grade level.

## 5. Discussion

The majority of the patient education materials on the AAST website has readability levels far above the reading comprehension level of the average patient and thus, may contain information that is too difficult to comprehend for a substantial portion of the patient population. Although the reading skills of the intended audience should be taken into consideration when patient education materials are developed, this must be weighed against the necessity of providing complete and accurate medical information. The readability of patient education materials can be improved by using simpler terms, shorter sentences, and plentiful illustrations (10, 26, 31, 32). According to the National Assessment of Adult Literacy, only 12% of adults have the health literacy skills needed to manage their health and prevent disease (33). Lower health literacy is associated with reduced health-related quality of life, reduced general health, and increased hospitalizations as well as complications. Together, these outcomes yield substantial increases in overall health-care costs. Therefore, improving health literacy may in turn improve patient’s outcomes.

### 5.1. Limitations of Study

This study had several potential limitations. We did not specifically assess the reading skills of the website’s readers as they may differ from the general population; however, trauma patients likely have similar or potentially lower rates of reading comprehension compared to the general public. A possible additional limitation is that the FKGL only evaluates text (i.e. not diagrams) and does not directly measure comprehensibility. Finally, although we only reviewed one society’s patient education materials, the sample is relevant as surgeons increasingly refer their patients to such professional websites (34-36).

### 5.2. Important Conclusion

This study found that the AAST patient education website currently contains information at a readability level too advanced for most patients to comprehend. Improving readability of patient education materials may improve patient understanding and thus, positively affect health outcomes. Moreover, a better-informed public would be a powerful ally in the efforts to increase trauma awareness, public funding, and injury prevention.
